# *DPYD* and *UGT1A1* genotyping to predict adverse events during first-line FOLFIRI or FOLFOXIRI plus bevacizumab in metastatic colorectal cancer

**DOI:** 10.18632/oncotarget.23559

**Published:** 2017-12-21

**Authors:** Chiara Cremolini, Marzia Del Re, Carlotta Antoniotti, Sara Lonardi, Francesca Bergamo, Fotios Loupakis, Beatrice Borelli, Federica Marmorino, Valentina Citi, Enrico Cortesi, Roberto Moretto, Monica Ronzoni, Gianluca Tomasello, Alberto Zaniboni, Patrizia Racca, Angela Buonadonna, Giacomo Allegrini, Vincenzo Ricci, Samantha Di Donato, Vittorina Zagonel, Luca Boni, Alfredo Falcone, Romano Danesi

**Affiliations:** ^1^ Unit of Medical Oncology 2, Azienda Ospedaliera-Universitaria Pisana, University of Pisa, Istituto Toscano Tumori, Pisa, Italy; ^2^ Clinical Pharmacology and Pharmacogenetics Unit, Department of Clinical and Experimental Medicine, University of Pisa, Pisa, Italy; ^3^ Oncologia Medica 1, Istituto Oncologico Veneto, Istituto di Ricovero e Cura a Carattere Scientifico (IRCCS), Padova, Italy; ^4^ Department of Medical Oncology, Policlinico Umberto I “Sapienza” University of Rome, Roma, Italy; ^5^ Medical Oncology Unit, Ospedale San Raffaele-IRCCS, Milano, Italy; ^6^ ASST di Cremona, Ospedale di Cremona, Cremona, Italy; ^7^ Fondazione Poliambulanza Hospital, Brescia, Italy; ^8^ SSD ColoRectal Unit-Dipartimento di Oncologia, AOU Città della Salute e della Scienza di Torino, Torino, Italy; ^9^ Medical Oncology Unit, CRO-National Cancer Institute, Aviano, Italy; ^10^ Medical Oncology Unit, Presidio Ospedaliero Felice Lotti, Pontedera, Italy; ^11^ Department of Oncology, S. Croce and Carle Teaching Hospital, Cuneo, Italy; ^12^ Medical Oncology Department, Nuovo Ospedale-Santo Stefano, Istituto Toscano Tumori, Prato, Italy; ^13^ Clinical Trial Coordinating Center, AOU Careggi, Istituto Toscano Tumori, Firenze, Italy

**Keywords:** DPYD, UGT1A1, 5-fluorouracil, irinotecan, toxicity

## Abstract

Our study addresses the issue of the clinical reliability of three candidate DPYD and one UGT single nucleotide polymorphisms in predicting 5-fluorouracil- and irinotecan-related adverse events. To this purpose, we took advantage of a large cohort of metastatic colorectal cancer patients treated with first-line 5-fluorouracil- and irinotecan-based chemotherapy regimens (i.e., FOLFIRI or FOLFOXIRI) plus bevacizumab in the randomized clinical trial TRIBE by GONO (clinicaltrials.gov: NCT00719797), in which adverse events were carefully and prospectively collected at each treatment cycle. Here we show that patients bearing *DPYD* c.1905+1G/A and c.2846A/T genotypes, together with UGT1A1*28 variant carriers, have an increased risk of experiencing clinically relevant toxicities, including hematological AEs and stomatitis. No carrier of the *DPYD* c.1679T>G minor allele was identified. Present results support the preemptive screening of mentioned *DPYD* and *UGT1A1* variants to identify patients at risk of clinically relevant 5-fluoruracil- and irinotecan-related AEs, in order to improve treatments’ safety through a “genotype-guided” approach.

## INTRODUCTION

5-fluorouracil (5-FU) and irinotecan, together with oxaliplatin, are the milestones of the first-line treatment of metastatic colorectal cancer (mCRC) [1]. Around 35–50% of patients treated with combination regimens including 5-FU and irinotecan experience unpredictable and sometimes clinically relevant treatment-related toxicities, mainly diarrhea, stomatitis and myelosuppression [2, 3]. Based on efficacy results of recent randomized trials [3–6], FOLFOXIRI (5-FU, oxaliplatin and irinotecan) plus bevacizumab is now recommended by all major guidelines as upfront regimen for selected mCRC patients [7, 8]. Although feasible, its use is associated with increased grade 3/4 neutropenia, diarrhea and stomatitis, so that tools able to predict the probability to develop potentially severe toxicities would be of major interest in order to better select candidate patients and to properly manage the treatment.

A substantial interindividual difference in the occurrence and/or seriousness of 5-FU- and irinotecan-related toxicities may be partially due to clinical factors, including age, sex and performance status [9]. Nonetheless, variability in individuals’ drug-metabolism may contribute as well [10, 11]. Indeed, deficiencies in two key enzymes involved in the first steps of the catabolic pathways of these two drugs, dihydropyrimidine dehydrogenase (DPD) and uridine diphosphate glucuronosyltransferases (UGT) 1A1 [12, 13], lead to increased exposure to the cytotoxic agents and their active metabolites with higher risk of related adverse events (AEs).

Most cases of DPD and UGT1A1 deficiency are attributable to germline polymorphisms in their encoding genes, leading to partially non-functional enzymes [10, 14].

Among more than 50 described allelic variants of *DPYD* [15], only a few of them have clinical relevance, resulting in the synthesis of non-functional or poorly functional enzymes, and thus exposing patients to an increased risk of 5-FU-related AEs [16]. To date, three *DPYD* variants, clearly affecting DPD activity, have been suggested with the highest level of evidence as predictors of severe toxicity from 5-FU: DPYD*2A (IVS14+1G>A, c.1905+1G>A or rs3918290), *DPYD* p.D949V (c.2846A>T or rs67376798) and DPYD*13 (p.I560S, c.1679T>G or rs55886062) [17–20]. All three variants have very low estimated frequencies of minor alleles in the Caucasian population (0.1 to 1.0%) [16, 17].

A variable number of TA repeats in the promoter region of *UGT1A1* affects its transcriptional efficiency [21, 22]. In particular, when compared with the wild-type six-repeat allele (UGT1A1*1), the seven-repeat variant (UGT1A1*28) is responsible for a dramatically reduced expression of UGT1A1, resulting in poor metabolism of the SN38 active metabolite of irinotecan and increased neutropenia and, to a lesser extent, diarrhea [13, 23].

The present study has been conceived with the purpose to evaluate the individual association of three *DPYD* single nucleotide polymorphisms (SNPs), whose relation with 5-FU-related toxicity is more robust [17], and of UGT1A1*28 variant with chemotherapy-related AEs experienced by patients treated with first-line FOLFOXIRI plus bevacizumab or FOLFIRI plus bevacizumab in the phase III TRIBE trial.

## RESULTS

Four hundred and forty-three (87%) out of 508 randomized patients were tested for *DPYD* and *UGT1A1* variants, and defined as “pharmacogenetic assessable population”. All patients received study treatments at planned dosages and, in the case of pre-specified AEs, treatment modifications were allowed according to study protocol. Main demographic and clinical characteristics at baseline did not differ between the pharmacogenetic assessable and the intention-to-treat population (Supplementary Table 1), as well as the incidence of treatment-related grade ≥ 3 AEs (Supplementary Table 2) [3]. In the pharmacogenetic assessable population, 225 patients (51%) experienced any grade ≥3 AE (overall AEs), with 102 (23%) and 170 (38%) patients reporting grade ≥3 gastrointestinal and hematological AEs, respectively. Most frequent chemotherapy-related toxicities included neutropenia (37%), diarrhea (15%), febrile neutropenia (8%) and stomatitis (7%).

Among investigated clinical characteristics, age, sex and treatment arm had a significant impact on the occurrence of grade 3 or greater treatment-related overall AEs in univariate analysis. Associations between clinical variables and AEs are shown in Table 1. Female gender and, to a lesser extent, worst ECOG performance status (PS) and older age were associated with a higher risk of grade ≥ 3 overall gastrointestinal AEs. As expected, patients in the FOLFOXIRI plus bevacizumab arm experienced more frequently grade ≥ 3 neutropenia, overall gastrointestinal and hematological AEs.

**Table 1:** Association of relevant clinical variables with grade ≥ 3 AEs

Grade ≥ 3 AEs	Age^a^		Sex (Male/Female)^b^		Treatment arm^c^		ECOG PS^d^	
	OR (95% CI)	*P* value	OR (95% CI)	*P* value	OR (95% CI)	*P* value	OR (95% CI)	*P* value
**Nausea**	1.08 (0.74–1.58)	0.52	3.93 (1.21–12.74)	0.02	0.96 (0.33–2.78)	0.94	5.23 (1.67–16.35)	0.004
**Vomit**	1.14 (0.81–1.59)	0.45	3.16 (1.16–8.58)	0.02	1.21 (0.47–3.12)	0.69	3.58 (1.22–10.56)	0.02
**Diarrhea**	1.32 (1.09–1.60)	0.005	1.31 (0.77–2.21)	0.32	1.58 (0.93–2.69)	0.09	1.44 (0.66–3.15)	0.36
**Stomatitis**	1.14 (0.88–1.49)	0.32	3.27 (1.49–7.17)	0.003	2.01 (0.92–4.40)	0.08	1.35 (0.45–4.06)	0.59
**Neutropenia**	1.11b (0.97–1.28)	0.14	1.58 (1.07–2.35)	0.02	3.44 (2.28–5.19)	< 0.001	0.58 (0.29–1.15)	0.12
**Febrile neutropenia**	1.01 (0.79–1.29)	0.92	1.47 (0.73–2.93)	0.28	1.15 (0.58–2.31)	0.69	1.12 (0.38–3.32)	0.84
**Thrombocytopenia**	1.24 (0.69–2.22)	0.47	0.75 (0.14–4.14)	0.74	12.82 (0.71–230.54)	0.08	1.73 (0.20–15.17)	0.62
**Anemia**	3.09 (1.16–8.18)	0.02	3.06 (0.55–16.88)	0.20	12.82 (0.71–230.54)	0.08	0.65 (0.04–11.97)	0.77
**Overall gastrointestinal AEs^e^**	1.27 (1.08–1.49)	0.004	2.00 (1.28–3.13)	0.002	1.59 (1.01–2.49)	0.04	1.92 (1.00–3.68)	0.05
**Overall hematological AEs^f^**	1.11 (0.97–1.27)	0.14	1.72 (1.16–2.54)	0.007	3.42 (2.27–5.13)	< 0.001	0.60 (0.31–1.18)	0.14
**Overall AEs^g^**	1.19 (1.04–1.36)	0.01	1.89 (1.28–2.78)	0.001	2.80 (1.90–4.11)	< 0.001	0.97 (0.52–1.78)	0.91

Abbreviations: OR, odds ratio; ECOG PS, Eastern Cooperative Oncology Group Performance Status.

^a^reported ORs refer to a quintile increase of the predictor variable; ^b^reported ORs refer to female vs male; c: reported ORs refer to arm receiving FOLFOXIRI plus bevacizumab vs arm receiving FOLFIRI plus bevacizumab; ^d^reported ORs refer to ECOG PS 1-2 vs 0; ^e^including nausea, vomit, diarrhea, stomatitis; ^f^including neutropenia, febrile neutropenia, thrombocytopenia, anemia; ^g^including neutropenia, febrile neutropenia, thrombocytopenia, anemia, nausea, vomit, diarrhea, stomatitis. *P* values in bold indicate statistical significance.

Four hundred and thirty-nine (99%) out of 443 patients were successfully genotyped for investigated *DPYD* variants (*DPYD* c.1905+1G>A; *DPYD* c.2846A>T; *DPYD* c.1679T>G) and 436 (98%) for UGT1A1*28 variant (Table 2).

**Table 2:** DPYD and UGT1A1 genotypes frequency in the pharmacogenetic assessable population

Variants genotyped	Treatment arm		Pharmacogenetic assessable population No. (%) *n* = 443
	Arm A FOLFIRI+bev< No. (%) *n* = 217	Arm B FOLFOXIRI+bev No. (%) *n* = 226	
***DPYD c*.1905+1G>A**			
**G/G**	213 (99.1%)	222 (98.7%)	435 (98.9%)
**G/A**	2 (0.9%)	3 (1.3%)	5 (1.1%)
**A/A**	0	0	0
**NE**	2	1	3
***DPYD c.*2846A>T**			
**A/A**	213 (99.1%)	222 (98.7%)	435 (98.9%)
**A/T**	2 (0.9%)	3 (1.3%)	5 (1.1%)
**T/T**	0	0	0
**NE**	2	1	3
***DPYD c.*1679T>G**			
**T/T**	215 (100%)	225 (100%)	440 (100%)
**T/G**	0	0	0
**G/G**	0	0	0
**NE**	2	1	3
UGT1A1^*^28			
^*^1/^*^1	74 (34.7%)	72 (32.4%)	146 (33.5%)
^*^1/^*^28	119 (55.9%)	132 (59.5%)	251 (57.6%)
^*^28/^*^28	20 (9.4%)	19 (8.6%)	39 (8.9%)
**NE**	4	3	7

FOLFIRI: fluorouracil, leucovorin, and irinotecan; FOLFOXIRI: fluorouracil, leucovorin, oxaliplatin and irinotecan; bev: bevacizumab; NE: not evaluable.

Among patients genotyped for *DPYD* variants, minor alleles for *DPYD* c.1905+1G>A and *DPYD* c.2846 A>T were found only in heterozygosis in 5 (1.1%) and 5 (1.1%) patients, respectively; no carriers of *DPYD* c.1679T>G minor allele were identified (Table 2). Allele frequencies for *DPYD* c.1905+1G>A, *DPYD* c.2846A>T and *DPYD* c.1679T>G were consistent with published data [16, 24] and were in Hardy-Weinberg equilibrium (χ^2^ test *p* value: 0.90). Out of 436 patients genotyped for *UGT1A1*, *1/*1, *1/*28 and *28/*28 genotypes were detected in 146 (33.5%), 251 (57.6%) and 39 (8.9%) cases, respectively (Table 2).

Additionally, as detailed in Supplementary Figure 1, seven patients showed the concomitant presence of *DPYD* and UGT1A1*28 minor variants. In particular, *DPYD* c.1905+1G/A variant was concomitantly found with *UGT1A1* *1/*28 genotype in three patients, and *DPYD* c.2846A/T was associated with *UGT1A1* *1/*28 and *28/*28 in three and one patient, respectively.

Eight (80%) out of 10 *DPYD* c.1905+1G/A (*n* = 5) or *DPYD* c.2846A/T (*n* = 5) carriers experienced at least one grade ≥ 3 AE during the treatment. Seven (70%) out of 10 patients bearing a *DPYD* variant allele had a grade ≥ 3 AE within the first four cycles of induction therapy (Supplementary Table 3) as compared to 166 (39%) out of 429 patients bearing *DPYD* c.1905+1G/G and *DPYD* c.2846A/A genotypes (*P* = 0.055). Most frequent AEs were neutropenia (70%), stomatitis (40%), diarrhea and febrile neutropenia (20%), and thrombocytopenia (10%).

Significant associations were identified between both *DPYD* c.1905+1G/A (OR, 9.69 [95% CI, 1.56–60.39]; *P* = 0.02) and *DPYD* c.2846A/T (OR, 9.69 [95% CI, 1.56–60.39]; *P* = 0.02) variants and grade ≥3 stomatitis (Supplementary Table 4) and between *DPYD* c.1905+1G/A and grade ≥ 3 thrombocytopenia (OR, 21.50 [95% CI, 2.02–228.16]; *P* = 0.01). In the multivariate model, including age, sex, treatment arm and ECOG PS, both *DPYD* c.1905+1G/A (OR, 17.32 [95% CI, 2.50–120.12]; *P* = 0.004) and *DPYD* c.2846A/T (OR, 14.11 [95% CI, 2.01–99.29]; *P* = 0.008) variants retained their association with grade ≥ 3 stomatitis; the association between *DPYD* c.1905+1G/A and thrombocytopenia was also confirmed (OR, 62.81 [95% CI, 4.41–895.12]; *P* = 0.002) (Table 3). Furthermore, at the multivariate analysis, *DPYD* c.1905+1G/A was significantly associated with a higher risk of anemia (OR, 41.26 [95% CI, 1.74–903.61]; *P* = 0.04) (Table 3).

**Table 3:** Multivariate analysis adjusted for age, sex, treatment arm and ECOG PS, testing association hypotheses of *DPYD* c.1905+1G>A and *DPYD* c.2846A>T genotypes with AEs

Grade ≥ 3 AEs	DPYD c.1905+1G>A^d^		DPYD c.2846A>T^e^		DPYD c.1905+1G>A and DPYD c.2846A>T^f^	
	OR [95% CI]	*P* value	OR [95% CI]	*P* value	OR [95% CI]	*P* value
**Nausea**	5.05 [0.20–128.63]	0.33	3.93 [0.15–104.06]	0.41	2.35 [0.12–46.78]	0.58
**Vomit**	3.51 [0.14–88.02]	0.44	2.60 [0.10–68.13]	0.57	1.58 [0.08–31.42]	0.77
**Diarrhea**	0.69 [0.03–16.63]	0.82	4.03 [0.65–25.12]	0.14	1.91 [0.42–8.79]	0.40
**Stomatitis**	17.32 [2.50–120.12]	**0.004**	14.11 [2.01–99.29]	**0.008**	16.95 [3.97–72.34]	**< 0.001**
**Neutropenia**	6.23 [0.75–51.52]	0.09	2.42 [0.37–15.95]	0.36	4.14 [1.01–16.95]	**0.05**
**Febrile neutropenia**	4.18 [0.53–33.22]	0.18	4.09 [0.52–32.33]	0.18	3.83 [0.83–17.72]	0.09
**Thrombocytopenia**	62.81 [4.41–895.12]	**0.002**	5.23 [0.20–138.80]	0.32	16.17 [1.93–135.85]	0.01
**Anemia**	41.26 [1.74–903.61]	**0.04**	4.96 [0.13–193.65]	0.39	4.81 [0.17–137.96]	0.36
**Overall**	3.67 [0.60–22.68]	0.16	5.52 [0.87–34.89]	0.07	4.59 [1.25–16.84]	**0.02**
**gastrointestinal AEs^a^ Overall**	5.96 [0.72–49.43]	0.10	2.31 [0.35–15.29]	0.39	3.98 [0.97–16.33]	**0.05**
**hematological AEs^b^ Overall AEs^c^**	3.65 [0.46–28.94]	0.22	3.12 [0.38–25.49]	0.29	3.89 [0.85–17.90]	0.08

AEs, adverse events; OR, odds ratio. a: including nausea, vomit, diarrhea, stomatitis; b: including neutropenia, febrile neutropenia, thrombocytopenia, anemia.; c: including neutropenia, febrile neutropenia, thrombocytopenia, anemia, nausea, vomit, diarrhea, stomatitis; d: reported ORs refer to DPYD c.1905+1G/A vs DPYD c.1905+1G/G carriers; e: reported ORs refer to DPYD c.2846A/T vs DPYD c.2846A/A carriers; f: reported ORs refer to DPYD c.1905+1G/A or DPYD c.2846A/T vs DPYD c.1905+1G/G and DPYD c.2846A/A carriers. *P* values in bold indicate statistical significance.

Patients bearing *DPYD* c.1905+1G/A or *DPYD* c.2846A/T variants (*n* = 10) had an increased risk of experiencing grade ≥ 3 overall hematological AEs (OR, 3.88 [95% CI, 0.99–15.23]; *P* = 0.05), neutropenia (OR, 4.12 [95% CI, 1.05–16.17]; *P* = 0.04), thrombocytopenia (OR, 9.42 [95% CI, 1.00–89.06]; *P* = 0.05) and stomatitis (OR, 10.33 [95% CI, 2.74–38.91]; *P <* 0.001), as compared to patients bearing *DPYD* c.1905+1G/G and *DPYD* c.2846A/A genotypes (*n* = 429) (Supplementary Table 4). These associations were confirmed in the multivariate analysis, where increased risk of developing overall gastrointestinal AEs was also reported (OR, 4.59 [95% CI, 1.25–16.84]; *P* = 0.02) (Table 3).

Among patients carrying UGT1A1*1/*28 or UGT1A1*28/*28 genotype, the incidence of grade ≥3 overall AEs was 136/251 (54%) and 24/39 (62%), respectively.

Patients bearing UGT1A1*28/*28 genotype experienced more frequently a grade ≥ 3 AE within the first four cycles of induction therapy (22 out of 39, 56.4%) as compared to those carrying UGT1A1*1/*28 (108 out of 251, 43.0%) and UGT1A1*1/*1 genotypes (43 out of 146, 29.5%) (*P* = 0.002).

As shown in Supplementary Table 5, a significant association was found between *UGT1A1* variants and neutropenia (*P* = 0.001) with higher risk for UGT1A1*28/*28 (OR, 3.75 [95% CI, 1.80–7.80]) than UGT1A1*1/*28 variants (OR, 1.66 [95% CI, 1.07–2.59]), both compared with the UGT1A1*1/*1 group. Consistent results were reported in the multivariable model (*P* = 0.001). *UGT1A1* variants were also associated with the overall risk of developing hematological and overall AEs, both in the uni- and multivariable models.

Patients carriers of *DPYD* c.1905+1G/A or *DPYD* c.2846A/T and/or UGT1A1*28/*28 genotypes (*n* = 48) had a higher risk of experiencing grade ≥ 3 overall AEs (OR, 1.89 [95% CI, 1.01–3.53]; *P* = 0.05), overall hematological AEs (OR, 2.79 [95% CI, 1.51–5.15]; *P* = 0.001), stomatitis (OR, 3.32 [95% CI, 1.39–7.94]; *P* = 0.007), neutropenia (OR, 2.98 [95% CI, 1.61–5.52]; *P<* 0.001) and febrile neutropenia (OR, 2.78 [95% CI, 1.18–6.54]; *P* = 0.02), compared to patients bearing *DPYD* c.1905+1G/G and *DPYD* c.2846A/A and UGT1A1*1/− genotypes (*n* = 387) (Supplementary Table 6). These associations were confirmed in the multivariable models (Supplementary Table 6).

The sensitivity and specificity of the combined evaluation of *DPYD* and *UGT1A1* genotypes as predictor of overall grade ≥ 3 AEs were 14% and 92%, respectively.

## DISCUSSION

In the last few years, a growing amount of mainly retrospective data suggested the role of *DPYD* and *UGT1A1* variants as potential risk factors for toxicities in patients treated with fluoropyrimidines (*i.e.*, 5-FU and capecitabine) and irinotecan, respectively [19, 23]. Nevertheless, since the current guidelines do not provide a firm consensus on the clinical validity of pretreatment *DPYD* and *UGT1A1* screening [7, 8], the implementation of these pharmacogenetic tests in the daily clinical practice remains a highly debated issue, especially regarding *DPYD* genotyping [25, 26]. The lack of evidence from prospective studies, together with some inconsistent results from retrospective series, in particular about *DPYD*, slowed a widespread consensus on these tests and the definition of conclusive recommendations on the implementation in the clinical practice of a “genotype-guided” dosing of cytotoxic agents.

Recently, this controversy acquired growing remark in the precision medicine scenario, and in the perspective of individualizing therapies and improving patients’ safety and clinical benefit. Indeed, the preventive identification of patients deemed at risk of developing clinically relevant 5-FU- and irinotecan-related AEs, able to heavily affect treatment feasibility, may represent a useful and rationale tool to drive the therapeutic decision-making process, as well as treatment management.

In the present work, we genotyped three *DPYD* and one *UGT1A1* variants in a large cohort of mCRC patients enrolled in the phase III TRIBE trial. With regard to *DPYD*, we focused on three SNPs whose relation with 5-FU-related toxicities is more robust [17], as recommended by a position paper shared by the Italian societies of Medical Oncology and Pharmacology (AIOM-SIF Working Group) [27].

In our opinion, the main strength point of this analysis lies in the availability of a large cohort of patients enrolled in a clinical trial with homogeneous baseline characteristics, including type of cancer, stage of disease, and line of treatment, and with a careful and uniform assessment of treatment-related AEs, performed at every cycle of therapy and graded according to NCI-CTCAE, version 3.0.

Through the analysis of 443 patients from TRIBE trial, we identified statistically significant associations between *DPYD* c.1905+1G/A and *DPYD* c.2846A/T genotypes and grade ≥ 3 hematological AEs and stomatitis, and between UGT1A1*28 variant and grade ≥ 3 hematological AEs, in particular neutropenia, regardless of the treatment arm. We were unable to assess any relation between *DPYD* c.1679T>G and 5-FU-related AEs, because none of our patients harbored a G allele, consistently with its low minor allele frequency (around 0.1% among Caucasians) [16]. In addition, we showed that taking into account both *DPYD* and *UGT1A1* genotypes allows predicting with high specificity grade ≥ 3 hematological AEs, including febrile neutropenia, and stomatitis. Furthermore, in *DPYD* and *UGT1A1* variant allele carriers we observed that grade ≥ 3 AEs occurred more frequently early during the treatment, within the first four cycles of therapy. These events were often clinically relevant, requiring dose modifications and/or delays in therapy administration as well as preventing treatment continuation in more serious cases.

In order to prevent the occurrence of treatment-related AEs in patients known to be carriers of *DPYD* or UGT1A1*28 variants, some reports recommended to adopt a reduced starting dose of 5-FU and irinotecan, respectively, to be then increased based on reported toxicities, while not affecting treatment efficacy [17, 28–32]. The proposed practical approach may be of crucial interest for an optimal and proper management of fluoropyrimidine- and irinotecan-based regimens (*i.e.*, FOLFOXIRI, FOLFIRI, XELIRI), supposing that the personalization of treatment starting doses according to *DPYD* and *UGT1A1* genotypes might contribute to decrease the incidence of some preventable AEs. The lack of planned *DPYD* and *UGT1A1* genotype-guided dose modifications in the TRIBE study clearly prevents us from deriving definitive conclusions about the effectiveness of the dose titration approach in reducing the incidence of AEs and improving treatment adherence, while not compromising its efficacy.

A potential contribution to fluoropyrimidines toxicity has been recently hypothesized for other *DPYD* variants, including c.2194G>A [20, 22]. However, the association of these variants with toxicity has not been consistently replicated in different series [33–35], so that prospective and well-powered studies able to further validate this association are urgently needed.

In conclusion, based on present results and consistent with literature data, our work supports the upfront test of *DPYD* c.1905+1G>A and c.2846A>T and UGT1A1*28 variants for the assessment of the risk of AEs in all mCRC patients candidate to first-line 5-FU- and irinotecan-containing regimens.

## MATERIALS AND METHODS

### Patients

TRIBE (TRIplet plus BEvacizumab) was a multicenter, randomized, phase III trial conducted by the Italian Cooperative GONO (Gruppo Oncologico Nord Ovest) group (NCT00719797). Five-hundred and eight unresectable mCRC patients, untreated for the metastatic disease were randomized to receive either FOLFOXIRI plus bevacizumab or FOLFIRI plus bevacizumab as initial treatment. Patients received up to 12 cycles of induction treatment according to randomization, followed by maintenance therapy with 5-FU and bevacizumab until evidence of disease progression in both arms. Full details of the TRIBE study have been previously published [3]. The emergence of AEs was biweekly monitored and graded according to National Cancer Institute-Common Toxicity Criteria for Adverse Events (NCI-CTCAE), version 3.0 [36].

Patients enrolled in the TRIBE trial, included in the safety population (*i.e.*, who had received at least one cycle of the assigned study treatment), providing their written informed consent to blood sampling and pharmacogenetic analyses were evaluated. The protocol was approved by local Ethics Committees at each participating site.

### Genotyping

One ml of whole blood were taken from all patients, stored in EDTA, and genomic DNA was extracted from 200 μl by the Biorobot EZ1 (Qiagen^®^, Valencia, CA, USA). The *DPYD* and *UGT1A1* variants were analysed by pyrosequencing method. DNA was amplified using the “Fluoropyrimidines response” and “Irinotecan response” kit (Diatech Pharmacogenomics^®^, Jesi, Italy) on a RotorGene TM6000 (Corbett Research^®^, Sydney, Australia), according to the manufacturer's instructions. The reaction products were run on a PyroMark Q69 ID system, and the results were analysed on the PyroMark Q24 1.0.9 software (Biotage^®^, Uppsala, Sweden).

### Statistical analysis

The study endpoints were the development of grade ≥ 3 gastrointestinal AEs, including nausea, vomit, diarrhea and stomatitis, and/or hematological AEs, including neutropenia, febrile neutropenia, thrombocytopenia and anemia. Logistic regression modeling was used to test the hypothesis of associations between the genotypes of each *DPYD* and *UGT1A1* polymorphism and the end points. Firth's penalized maximum likelihood estimation was used to avoid modeling separability issues. Associations of tested polymorphisms were assessed using the Wald chi-square test. Using a sample size of 440, associations with an OR equal to 5 for an overall grade 3 or greater toxicity-related allele of 3% could be detected with an α = 0.05 and a power of 0.80. To account for potential confounding factors, multivariate models were used. A set of 4 relevant clinical variables (sex, age, treatment arm and ECOG PS) was used (Table 1). The frequency of each *DPYD* variant was compared to the published frequencies in dbSNP [15] and tested for departure from Hardy-Weinberg equilibrium. Sensitivity and specificity were calculated for the combined assessment of *DPYD* and *UGT1A1* genotypes.

All statistical tests were two-sided and *P* values of 0.05 or less were considered statistically significant.

## SUPPLEMENTARY MATERIALS FIGURE AND TABLES









## References

[1] Cremolini C, Schirripa M, Antoniotti C, Moretto R, Salvatore L, Masi G, Falcone A, Loupakis F (2015). First-line chemotherapy for mCRC-a review and evidence-based algorithm. Nat Rev Clin Oncol.

[2] Falcone A, Ricci S, Brunetti I, Pfanner E, Allegrini G, Barbara C, Crinò L, Benedetti G, Evangelista W, Fanchini L, Cortesi E, Picone V, Vitello S, Gruppo Oncologico Nord Ovest (2007). Phase III trial of infusional fluorouracil, leucovorin, oxaliplatin, and irinotecan (FOLFOXIRI) compared with infusional fluorouracil, leucovorin, and irinotecan (FOLFIRI) as first-line treatment for metastatic colorectal cancer: the Gruppo Oncologico Nord Ovest. J Clin Oncol.

[3] Loupakis F, Cremolini C, Masi G, Lonardi S, Zagonel V, Salvatore L, Cortesi E, Tomasello G, Ronzoni M, Spadi R, Zaniboni A, Tonini G, Buonadonna A (2014). Initial therapy with FOLFOXIRI and bevacizumab for metastatic colorectal cancer. N Engl J Med.

[4] Bendell J, Tan B, Reeves JA, Xiong H, Somer B, Lenz HJ, Hochster H, Scappaticci F, Sommer N, Day B, Hurwitz H (2016). Overall response rate (ORR) in STEAM, a randomized, open-label, phase 2 trial of sequential and concurrent FOLFOXIRI-bevacizumab (BEV) vs FOLFOX-BEV for the first-line (1L) treatment (tx) of patients (pts) with metastatic colorectal cancer (mCRC). J Clin Oncol.

[5] Gruenberger T, Bridgewater J, Chau I, Garcia Alfonso P, Rivoire M, Mudan S, Lasserre S, Hermann F, Waterkamp D, Adam R (2015). Bevacizumab plus mFOLFOX-6 or FOLFOXIRI in patients with initially unresectable liver metastases from colorectal cancer: the OLIVIA multinational randomised phase II trial. Ann Oncol.

[6] Schmoll H, Garlipp B, Junghanss C, Leithaeuser M, Vogel A, Schaefers M, Kaiser U, Hoeffkes HG, Florschutz A, Russel J, Kanzler S, Edelmann T, Forstbauer H (2017). CHARTA: FOLFOX+bevacizumab +/− irinotecan in advanced colorectal cancer (CRC)-Final results of the randomized phase II trial AIO (KRK 0209). J Clin Oncol.

[7] Van Cutsem E, Cervantes A, Adam R, Sobrero A, Van Krieken JH, Aderka D, Aranda Aguilar E, Bardelli A, Benson A, Bodoky G, Ciardiello F, D'Hoore A, Diaz-Rubio E (2016). ESMO consensus guidelines for the management of patients with metastatic colorectal cancer. Ann Oncol.

[8] National Comprehensive Cancer Network (2017). NCCN Clinical Practice Guidelines in Oncology — Colon Cancer; Version 1.2017. https://www.nccn.org/professionals/physician_gls/pdf/colon.pdf.

[9] Stein BN, Petrelli NJ, Douglass HO, Driscoll DL, Arcangeli G, Meropol NJ (1995). Age and sex are independent predictors of 5-fluorouracil toxicity. Analysis of a large scale phase III trial. Cancer.

[10] van Kuilenburg AB (2004). Dihydropyrimidine dehydrogenase and the efficacy and toxicity of 5-fluorouracil. Eur J Cancer.

[11] Gupta E, Lestingi TM, Mick R, Ramirez J, Vokes EE, Ratain MJ (1994). Metabolic fate of irinotecan in humans: correlation of glucuronidation with diarrhea. Cancer Res.

[12] Amstutz U, Froehlich TK, Largiader CR (2011). Dihydropyrimidine dehydrogenase gene as a major predictor of severe 5-fluorouracil toxicity. Pharmacogenomics.

[13] Liu X, Cheng D, Kuang Q, Liu G, Xu W (2014). Association of UGT1A1*28 polymorphisms with irinotecan-induced toxicities in colorectal cancer: a meta-analysis in Caucasians. Pharmacogenomics J.

[14] Beutler E, Gelbart T, Demina A (1998). Racial variability in the UDP-glucuronosyltransferase 1 (UGT1A1) promoter: a balanced polymorphism for regulation of bilirubin metabolism?. Proc Natl Acad Sci U S A.

[15] National Center for Biotechnology Information Database of single nucleotide polymorphisms (dbSNP). https://www.ncbi.nlm.nih.gov/SNP/.

[16] Meulendijks D, Cats A, Beijnen JH, Schellens JH (2016). Improving safety of fluoropyrimidine chemotherapy by individualizing treatment based on dihydropyrimidine dehydrogenase activity - Ready for clinical practice?. Cancer Treat Rev.

[17] Caudle KE, Thorn CF, Klein TE, Swen JJ, McLeod HL, Diasio RB, Schwab M (2013). Clinical Pharmacogenetics Implementation Consortium guidelines for dihydropyrimidine dehydrogenase genotype and fluoropyrimidine dosing. Clin Pharmacol Ther.

[18] Terrazzino S, Cargnin S, Del Re M, Danesi R, Canonico PL, Genazzani AA (2013). DPYD IVS14+1G>A and 2846A>T genotyping for the prediction of severe fluoropyrimidine-related toxicity: a meta-analysis. Pharmacogenomics.

[19] Meulendijks D, Henricks LM, Sonke GS, Deenen MJ, Froehlich TK, Amstutz U, Largiader CR, Jennings BA, Marinaki AM, Sanderson JD, Kleibl Z, Kleiblova P, Schwab M (2015). Clinical relevance of DPYD variants c.1679T>G, c.1236G>A/HapB3, and c.1601G>A as predictors of severe fluoropyrimidine-associated toxicity: a systematic review and meta-analysis of individual patient data. Lancet Oncol.

[20] Boige V, Vincent M, Alexandre P, Tejpar S, Landolfi S, Le Malicot K, Greil R, Cuyle PJ, Yilmaz M, Faroux R, Matzdorff A, Salazar R, Lepage C (2016). DPYD Genotyping to Predict Adverse Events Following Treatment With Flourouracil-Based Adjuvant Chemotherapy in Patients With Stage III Colon Cancer: A Secondary Analysis of the PETACC-8 Randomized Clinical Trial. JAMA Oncol.

[21] Innocenti F, Undevia SD, Iyer L, Chen PX, Das S, Kocherginsky M, Karrison T, Janisch L, Ramirez J, Rudin CM, Vokes EE, Ratain MJ (2004). Genetic variants in the UDP-glucuronosyltransferase 1A1 gene predict the risk of severe neutropenia of irinotecan. J Clin Oncol.

[22] Li M, Wang Z, Guo J, Liu J, Li C, Liu L, Shi H, Liu L, Li H, Xie C, Zhang X, Sun W, Fang S, Bi X (2014). Clinical significance of UGT1A1 gene polymorphisms on irinotecan-based regimens as the treatment in metastatic colorectal cancer. Onco Targets Ther.

[23] Campbell JM, Stephenson MD, Bateman E, Peters MD, Keefe DM, Bowen JM (2017). Irinotecan-induced toxicity pharmacogenetics: an umbrella review of systematic reviews and meta-analyses. Pharmacogenomics J.

[24] Lee AM, Shi Q, Pavey E, Alberts SR, Sargent DJ, Sinicrope FA, Berenberg JL, Goldberg RM, Diasio RB (2014). DPYD variants as predictors of 5-fluorouracil toxicity in adjuvant colon cancer treatment (NCCTG N0147). Natl Cancer Inst.

[25] Deenen MJ, Meulendijks D (2017). Recommendation on testing for dihydropyrimidine dehydrogenase deficiency in the ESMO consensus guidelines for the management of patients with metastatic colorectal cancer. Ann Oncol.

[26] Danesi R, Del Re M, Ciccolini J, Schellens JH, Schwab M, van Schaik RH, van Kuilenburg AB (2017). Prevention of fluoropyrimidine toxicity: do we still have to try our patient's luck?. Ann Oncol.

[27] AIOM-SIF Working Group (2015). Recommendations for pharmacogenetic analyses. http://www.aiom.it/C_Common/Download.asp?file=/$Site$/files/doc/documenti_scientifici/2015_aiom-sif_analisi_farmacogenetiche.pdf.

[28] Deenen MJ, Meulendijks D, Cats A, Sechterberger MK, Severens JL, Boot H, Smits PH, Rosing H, Mandigers CM, Soesan M, Beijnen JH, Schellens JH (2016). Upfront Genotyping of DPYD*2A to Individualize Fluoropyrimidine Therapy: A Safety and Cost Analysis. J Clin Oncol.

[29] Innocenti F, Schilsky RL, Ramirez J, Janisch L, Undevia S, House LK, Das S, Wu K, Turcich M, Marsh R, Karrison T, Maitland ML, Salgia R, Ratain MJ (2014). Dose-finding and pharmacokinetic study to optimize the dosing of irinotecan according to the UGT1A1 genotype of patients with cancer. J Clin Oncol.

[30] O'Dwyer PJ, Catalano RB (2006). Uridine diphosphate glucuronosyltransferase (UGT) 1A1 and irinotecan: practical pharmacogenomics arrives in cancer therapy. J Clin Oncol.

[31] Innocenti F, Vokes EE, Ratain MJ (2006). Irinogenetics: what is the right star?. J Clin Oncol.

[32] Launay M, Dahan L, Duval M, Rodallec A, Milano G, Duluc M, Lacarelle B, Ciccolini J, Seitz JF (2016). Beating the odds: efficacy and toxicity of dihydropyrimidine dehydrogenase-driven adaptive dosing of 5-FU in patients with digestive cancer. Br J Clin Pharmacol.

[33] Deenen MJ, Tol J, Burylo AM, Doodeman VD, de Boer A, Vincent A, Guchelaar HJ, Smits PH, Beijnen JH, Punt CJ, Schellens JH, Cats A (2011). Relationship between single nucleotide polymorphisms and haplotypes in DPYD and toxicity and efficacy of capecitabine in advanced colorectal cancer. Clin Cancer Res.

[34] Rosmarin D, Palles C, Church D, Domingo E, Jones A, Johnstone E, Wang H, Love S, Julier P, Scudder C, Nicholson G, Gonzalez-Neira A, Martin M (2014). Genetic markers of toxicity from capecitabine and other fluorouracil-based regimens: investigation in the QUASAR2 study, systematic review, and meta-analysis. J Clin Oncol.

[35] Schwab M, Zanger UM, Marx C, Schaeffeler E, Klein K, Dippon J, Kerb R, Blievernicht J, Fischer J, Hofmann U, Bokemeyer C, Eichelbaum M, German 5-FU Toxicity Study Group (2008). Role of genetic and nongenetic factors for fluorouracil treatment-related severe toxicity: a prospective clinical trial by the German 5-FU Toxicity Study Group. J Clin Oncol.

[36] National Cancer Institute-Common Terminology Criteria for Adverse Events v3.0 (CTCAE) (2006). https://ctep.cancer.gov/protocoldevelopment/electronic_applications/docs/ctcaev3.pdf.

