# Supplementary material for: DPYD and UGT1A1 genotyping to predict adverse events during first-line FOLFIRI or FOLFOXIRI plus bevacizumab in metastatic colorectal cancer
Source: Oncotarget. 2017 Dec 21;9(8):7859–66. doi: 10.18632/oncotarget.23559 (PMC5814264; doi:10.18632/oncotarget.23559)
Supplement: Supplementary file 1 [file oncotarget-09-7859-s001.pdf]

DPYD and *UGT1A1* genotyping to predict adverse events during first-line FOLFIRI or FOLFOXIRI plus bevacizumab in metastatic colorectal cancer

SUPPLEMENTARY MATERIALS

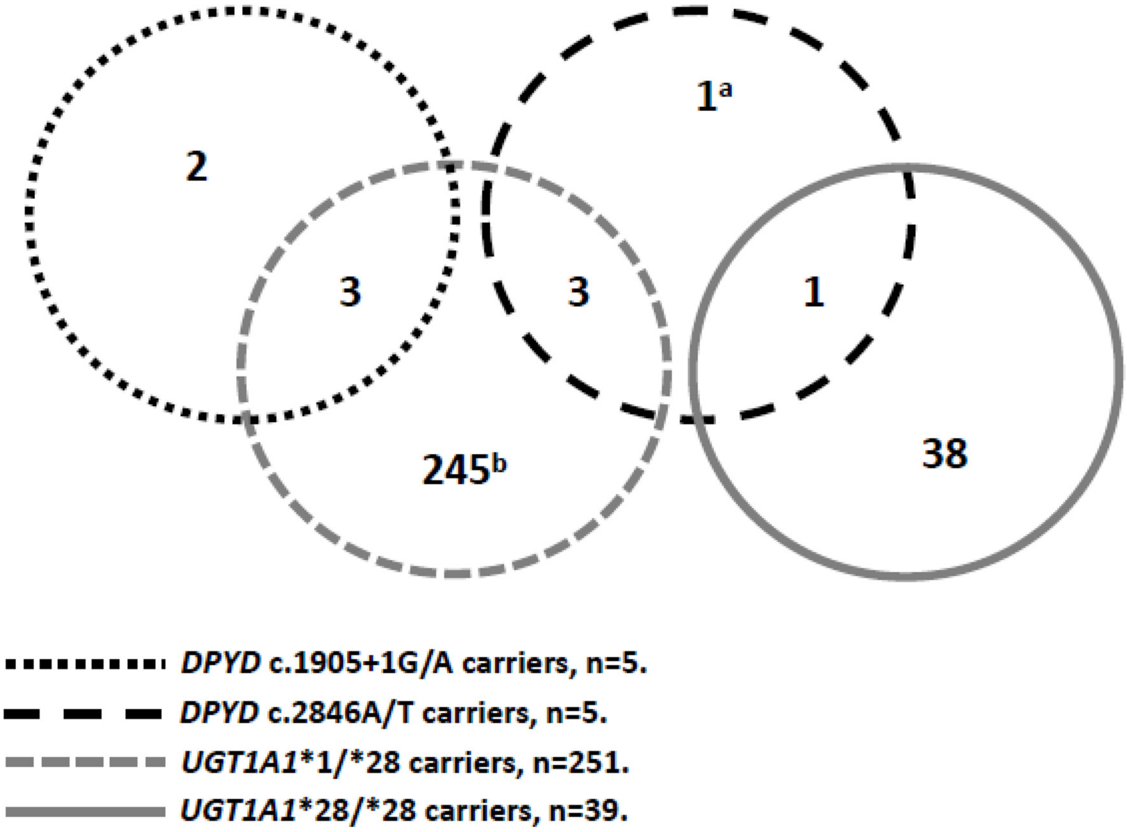

Supplementary Figure 1: Graphical representation of the distribution of the DPYD and UGT1A1 minor variants.

Supplementary Table 1: Main demographic and clinical characteristics of the pharmacogenetic assessable and intention-to-treat (ITT) populations

Characteristic	Pharmacogenetic assessable population (*n* pts= 443) Treatment arm		TRIBE ITT population (n pts= 508) Treatment arm	
	**Arm A FOLFIRI+bev (*n* = 217)**	**Arm B FOLFOXIRI+bev (*n* = 226)**	**Arm A FOLFIRI+bev (*n* = 256)**	**Arm B FOLFOXIRI+bev (*n* = 252)**
Median Age (range)	60 years (29–75)	61 years (30–75)	60 years (29–75)	61 years (29–75)
Sex, No. (%)				
Male	132 (61)	133 (59)	156 (61)	150 (60)
Female	85 (39)	93 (41)	100 (39)	102 (40)
ECOG PS, No. (%)				
0	195 (90)	201 (89)	229 (89)	227 (90)
1-2	22 (10)	25 (11)	27 (11)	25 (10)
Synchronous Metastases, No. (%)				
Yes	178 (82)	179 (79)	207 (81)	197 (78)
No	39 (18)	47 (21)	49 (19)	55 (22)
Prior Adjuvant chemotherapy, No. (%)				
Yes	26 (12)	27 (12)	33 (13)	33 (13)
No	191 (88)	199 (88)	223 (87)	219 (87)
Primary Tumor Site, No. (%)				
Right	54 (25)	77 (34)	61 (24)	88 (35)
Left or Rectum	150 (69)	140 (62)	179 (70)	152 (60)
Missing data	13 (6)	9 (4)	16 (6)	12 (5)
Liver Only Disease, No. (%)				
Yes	39 (18)	50 (22)	46 (18)	59 (23)
No	178 (82)	176 (78)	210 (82)	193 (77)
Resected Primary Tumor, No. (%)				
Yes	139 (64)	154 (68)	167 (65)	175 (69)
No	78 (36)	72 (32)	89 (35)	77 (31)

FOLFIRI: fluorouracil, leucovorin, and irinotecan; FOLFOXIRI: fluorouracil, leucovorin, oxaliplatin and irinotecan; bev: bevacizumab; ECOG PS, Eastern Cooperative Oncology Group Performance Status. Data presented as n (%) unless otherwise noted.

Supplementary Table 2: Treatment-related grade ≥ 3 AEs in the pharmacogenetic assessable population

AEs, No (%)	Treatment arm		Overall population<br>*n* = 443
	Arm A<br>FOLFIRI+bev<br>(*n* = 217)	Arm B<br>FOLFOXIRI+bev<br>(*n* = 226)	
Nausea	7 (3)	7 (3)	14 (3)
Vomit	8 (4)	10 (4)	18 (4)
Diarrhea	26 (12)	40 (18)	66 (15)
Stomatitis	10 (5)	20 (9)	30 (7)
Neutropenia	49 (23)	114 (50)	163 (37)
Febrile neutropenia	16 (7)	20 (9)	36 (8)
Thrombocytopenia	0	6 (3)	6 (1)
Anemia	0	6 (3)	6 (1)
Overall Gastrointestinal AEs[a]	41 (19)	61 (27)	102 (23)
Overall Hematological AEs[b]	52 (24)	118 (52)	170 (38)
Overall AEsc	82 (38)	143 (63)	225 (51)

AEs: adverse events; FOLFIRI: fluorouracil, leucovorin, and irinotecan; FOLFOXIRI: fluorouracil, leucovorin, oxaliplatin and irinotecan; bev: bevacizumab.

[a]: including nausea, vomit, diarrhea, stomatitis; [b]: including neutropenia, febrile neutropenia, thrombocytopenia, anemia.; [c]: including neutropenia, febrile neutropenia, thrombocytopenia, anemia, nausea, vomit, diarrhea, stomatitis. Data presented as n (%) unless otherwise noted.

Supplementary Table 3: Grade ≥ 3 AEs experienced by carriers of *DPYD* c.1905+1G>A or *DPYD* c.2846 A>T variants

Pt code	DPYD genotype	Treatment arm	Type of grade ≥ 3 AE	Time of occurrence of grade ≥ 3 AE
059	c.1905+1G/A	FOLFIRI+bev	G3 neutropenia	12th induction cycle
087	c.1905+1G/A	FOLFOXIRI+bev	None	-
106	c.1905+1G/A	FOLFOXIRI+bev	G4 neutropenia and thrombocytopenia; G3 stomatitis. Fatal.	1st induction cycle
115	c.2846A/T	FOLFOXIRI+bev	None	-
146	c.1905+1G/A	FOLFOXIRI+bev	G3 neutropenia; febrile neutropenia	2nd induction cycle
			G4 stomatitis and G3 neutropenia	3rd induction cycle
190	c.2846A/T	FOLFOXIRI+bev	G3 neutropenia	2nd induction cycle
252	c.2846A/T	FOLFIRI+bev	G3 diarrhea	4th induction cycle
322	c.2846A/T	FOLFOXIRI+bev	G4 diarrhea and neutropenia; febrile neutropenia	1st induction cycle
			G3 stomatitis and diarrhea	5th maintenance cycle
396	c.1905+1G/A	FOLFIRI+bev	G3 neutropenia	3rd induction cycle
406	c.2846A/T	FOLFIRI+bev	G4 neutropenia	1st induction cycle
			G3 stomatitis	5th induction cycle

Abbreviations: AEs: adverse events; FOLFIRI: fluorouracil, leucovorin, and irinotecan; FOLFOXIRI: fluorouracil, leucovorin, oxaliplatin and irinotecan; bev: bevacizumab.

Supplementary Table 4: Univariate analyses testing association hypotheses of *DPYD* c.1905+1G>A and *DPYD* c.2846A>T variants and Grade ≥3 AEs. See_Supplementary_Table 4

Supplementary Table 5: Univariate and multivariate analyses testing association hypotheses of *UGT1A1* variants with AEs. See_Supplementary_Table 5

Supplementary Table 6: Univariate and multivariate analyses testing association hypotheses of *DPYD* c.1905+1G>A and *DPYD* c.2846A>T and *UGT1A1* variants with AEs. See_Supplementary_ Table 6
