# Supplementary material for: DPYD and UGT1A1 genotyping to predict adverse events during first-line FOLFIRI or FOLFOXIRI plus bevacizumab in metastatic colorectal cancer
Source: Oncotarget. 2017 Dec 21;9(8):7859–66. doi: 10.18632/oncotarget.23559 (PMC5814264; doi:10.18632/oncotarget.23559)
Supplement: Supplementary file 2 [file oncotarget-09-7859-s002.docx]

**Supplementary Table 4. Univariate analyses testing association hypotheses of *DPYD* c.1905+1G>A and *DPYD* c.2846A>T variants and Grade ≥3 AEs.**

|  |  |  |  |  |  |  | ***DPYD* c.1905+1G>A^d^** | | | | |  |  |  |  |  |  |  | ***DPYD* c.2846A>T^e^** | | | | |  |  |  |  | ***DPYD* c.1905+1G>A and *DPYD* c.2846A>T^f^** | | | | | | | | | |  |  |
| --- | --- | --- | --- | --- | --- | --- | --- | --- | --- | --- | --- | --- | --- | --- | --- | --- | --- | --- | --- | --- | --- | --- | --- | --- | --- | --- | --- | --- | --- | --- | --- | --- | --- | --- | --- | --- | --- | --- | --- |
|  |  |  |  | **G/G** |  |  | **G/A** |  |  |  |  |  |  |  |  | **A/A** |  |  | **A/T** |  |  |  |  |  |  |  |  | **G/G and** |  |  | **G/A or A/T** |  |  |  |  |  |  |  |  |
|  | **Grade ≥3 AEs** |  |  |  |  |  |  |  |  |  |  |  |  |  |  |  |  |  |  |  |  |  |  |  |  |  |  | **A/A** |  |  |  |  |  |  |  |  |  |  |  |
|  |  |  |  | **carriers,** |  |  | **carriers,** |  |  | **OR** |  |  |  |  |  | **carriers,** |  |  | **carriers,** |  |  | **OR** |  |  |  |  |  |  |  |  | **carriers,** |  |  | **OR** |  |  |  |  |  |
|  |  |  |  |  |  |  |  |  |  |  |  |  | ***P* value** |  |  |  |  |  |  |  |  |  |  |  | ***P* value** |  |  | **carriers,** |  |  |  |  |  |  |  |  | ***P* value** |  |  |
|  |  |  |  | **no. (%)** |  |  | **no. (%)** |  |  | **[95% CI]** |  |  |  |  |  | **no. (%)** |  |  | **no. (%)** |  |  | **[95% CI]** |  |  |  |  |  |  |  |  | **no. (%)** |  |  | **[95% CI]** |  |  |  |  |  |
|  |  |  |  |  |  |  |  |  |  |  |  |  |  |  |  |  |  |  |  |  |  |  |  |  |  |  |  | **no. (%)** |  |  |  |  |  |  |  |  |  |  |  |
|  |  |  |  | **n=435** |  |  | **n=5** |  |  |  |  |  |  |  |  | **n=435** |  |  | **n=5** |  |  |  |  |  |  |  |  |  |  |  | **n=10** |  |  |  |  |  |  |  |  |
|  |  |  |  |  |  |  |  |  |  |  |  |  |  |  |  |  |  |  |  |  |  |  |  |  |  |  |  | **n=429** |  |  |  |  |  |  |  |  |  |  |  |
|  |  |  |  |  |  |  |  |  |  |  |  |  |  |  |  |  |  |  |  |  |  |  |  |  |  |  |  |  |  |  |  |  |  |  |  |  |  |  |  |
|  | **Nausea** | | 14 (3%) | |  | 0 (0%) | |  | 2.64 | |  | 0.55 | |  | 14 (3%) | |  | 0 (0%) | |  | 2.64 | |  | 0.55 | |  | 14 (3%) | |  | 0 (0%) | |  | 1.37 | |  | 0.84 | |  |  |
|  |  |  |  |  |  |  |  |  | [0.11-65.78] | |  |  |  |  |  |  |  |  |  |  | [0.11-65.78] | |  |  |  |  |  |  |  |  |  |  | [0.07-27.99] | |  |  |  |  |  |
|  |  |  |  |  |  |  |  |  |  |  |  |  |  |  |  |  |  |  |  |  |  |  |  |  |  |  |  |  |  |  |  |  |  |  |  |  |  |  |  |
|  | **Vomit** | | 18 (4%) | |  | 0 (0%) | |  | 2.05 | |  | 0.66 | |  | 18 (4%) | |  | 0 (0%) | |  | 2.05 | |  | 0.66 | |  | 18 (4%) | |  | 0 (0%) | |  | 1.06 | |  | 0.97 | |  |  |
|  |  |  |  |  |  |  |  |  | [0.08-50.60] | |  |  |  |  |  |  |  |  |  |  | [0.08-50.60] | |  |  |  |  |  |  |  |  |  |  | [0.05-21.53] | |  |  |  |  |  |
|  |  |  |  |  |  |  |  |  |  |  |  |  |  |  |  |  |  |  |  |  |  |  |  |  |  |  |  |  |  |  |  |  |  |  |  |  |  |  |  |
|  | **Diarrhea** | | 66 (15%) | |  | 0 (0%) | |  | 0.51 | |  | 0.67 | |  | 64 (15%) | |  | 2 (40%) | |  | 3.87 | |  | 0.14 | |  | 64 (15%) | |  | 2 (20%) | |  | 1.43 | |  | 0.66 | |  |  |
|  |  |  |  |  |  |  |  |  | [0.02-12.17] | |  |  |  |  |  |  |  |  |  |  | [0.63-23.59] | |  |  |  |  |  |  |  |  |  |  | [0.27-6.87] | |  |  |  |  |  |
|  |  |  |  |  |  |  |  |  |  |  |  |  |  |  |  |  |  |  |  |  |  |  |  |  |  |  |  |  |  |  |  |  |  |  |  |  |  |  |  |
|  | **Stomatitis** | | 28 (6%) | |  | 2 (40%) | |  | 9.69 | |  | **0.02** | |  | 28 (6%) | |  | 2 (40%) | |  | 9.69 | |  | **0.02** | |  | 26 (6%) | |  | 4 (40%) | |  | 10.33 | |  | **<0.001** | |  |  |
|  |  |  |  |  |  |  |  |  | [1.56-60.39] | |  |  |  |  |  |  |  |  |  |  | [1.56-60.39] | |  |  |  |  |  |  |  |  |  |  | [2.74-38.91] | |  |  |  |  |  |
|  |  |  |  |  |  |  |  |  |  |  |  |  |  |  |  |  |  |  |  |  |  |  |  |  |  |  |  |  |  |  |  |  |  |  |  |  |  |  |  |
|  | **Neutropenia** | | 158 (36%) | |  | 4 (80%) | |  | 7.00 | |  | 0.08 | |  | 159 (37%) | |  | 3 (60%) | |  | 2.60 | |  | 0.30 | |  | 155 (36%) | |  | 7 (70%) | |  | 4.12 | |  | **0.04** | |  |  |
|  |  |  |  |  |  |  |  |  | [0.78-63.14] | |  |  |  |  |  |  |  |  |  |  | [0.43-15.74] | |  |  |  |  |  |  |  |  |  |  | [1.05-16.17] | |  |  |  |  |  |
|  |  |  |  |  |  |  |  |  |  |  |  |  |  |  |  |  |  |  |  |  |  |  |  |  |  |  |  |  |  |  |  |  |  |  |  |  |  |  |  |
|  | **Febrile neutropenia** | | 34 (8%) | |  | 1 (20%) | |  | 2.95 | |  | 0.34 | |  | 33 | |  | 1 (20%) | |  | 3.05 | |  | 0.33 | |  | 32 (7%) | |  | 2 (20%) | |  | 3.10 | |  | 0.16 | |  |  |
|  |  |  |  |  |  |  |  |  | [0.32-27.12] | |  |  |  |  | (8%) | |  |  |  |  | [0.33-28.04] | |  |  |  |  |  |  |  |  |  |  | [0.63-15.22] | |  |  |  |  |  |
|  |  |  |  |  |  |  |  |  |  |  |  |  |  |  |  |  |  |  |  |  |  |  |  |  |  |  |  |  |  |  |  |  |  |  |  |  |  |  |  |
|  |  |  |  |  |  |  |  |  | 21.50 | |  |  |  |  |  |  |  |  |  |  | 6.00 | |  |  |  |  |  |  |  |  |  |  | 9.42 | |  |  |  |  |  |
|  | **Thrombocytopenia** | | 5 (1%) | |  | 1 (20%) | |  | [2.02- | |  | **0.01** | |  | 6 (1%) | |  | 0 (0%) | |  | [0.23- | |  | 0.28 | |  | 5 (1%) | |  | 1 (10%) | |  |  |  |  | **0.05** | |  |  |
|  |  |  |  |  |  |  |  |  |  |  |  |  |  |  |  |  |  |  |  |  |  |  |  |  |  |  |  |  |  |  |  |  | [1.00-89.06] | |  |  |  |  |  |
|  |  |  |  |  |  |  |  |  | 228.16] | |  |  |  |  |  |  |  |  |  |  | 157.23] | |  |  |  |  |  |  |  |  |  |  |  |  |  |  |  |  |  |
|  |  |  |  |  |  |  |  |  |  |  |  |  |  |  |  |  |  |  |  |  |  |  |  |  |  |  |  |  |  |  |  |  |  |  |  |  |  |  |  |
|  |  |  |  |  |  |  |  |  | 6.00 | |  |  |  |  |  |  |  |  |  |  | 6.00 | |  |  |  |  |  |  |  |  |  |  | 3.10 | |  |  |  |  |  |
|  | **Anemia** | | 6 (1%) | |  | 0 (0%) | |  | [0.23- | |  | 0.28 | |  | 6 (1%) | |  | 0 (0%) | |  | [0.23- | |  | 0.28 | |  | 6 (1%) | |  | 0 (0%) | |  |  |  |  | 0.47 | |  |  |
|  |  |  |  |  |  |  |  |  |  |  |  |  |  |  |  |  |  |  |  |  |  |  |  |  |  |  |  |  |  |  |  |  | [0.14-67.15] | |  |  |  |  |  |
|  |  |  |  |  |  |  |  |  | 157.23] | |  |  |  |  |  |  |  |  |  |  | 157.23] | |  |  |  |  |  |  |  |  |  |  |  |  |  |  |  |  |  |
|  |  |  |  |  |  |  |  |  |  |  |  |  |  |  |  |  |  |  |  |  |  |  |  |  |  |  |  |  |  |  |  |  |  |  |  |  |  |  |  |
|  | **Overall gastrointestinal** | | 100 (23%) | |  | 2 (40%) | |  | 2.23 | |  | 0.38 | |  | 99 (23%) | |  | 3 (60%) | |  | 5.10 | |  | 0.08 | |  | 97 (23%) | |  | 5 (50%) | |  | 3.42 | |  | 0.06 | |  |  |
|  | **AEs^a^** | |  |  |  |  |  |  | [0.37-13.55] | |  |  |  |  |  |  |  |  |  |  | [0.84-30.89] | |  |  |  |  |  |  |  |  |  |  | [0.97-12.07] | |  |  |  |  |  |
|  | **Overall hematological** | | 165 (38%) | |  | 4 (80%) | |  | 6.55 | |  | 0.09 | |  | 165 (38%) | |  | 3 (60%) | |  | 2.45 | |  | 0.33 | |  | 161 (38%) | |  | 7 (70%) | |  | 3.88 | |  | **0.05** | |  |  |
|  | **AEs^b^** | |  |  |  |  |  |  | [0.73-59.06] | |  |  |  |  |  |  |  |  |  |  | [0.41-14.84] | |  |  |  |  |  |  |  |  |  |  | [0.99-15.23] | |  |  |  |  |  |
|  | **Overall AEs^c^** | | 220 (51%) | |  | 4 (80%) | |  | 3.91 | |  | 0.22 | |  | 219 (50%) | |  | 4 (80%) | |  | 3.95 | |  | 0.22 | |  | 215 (50%) | |  | 8 (80%) | |  | 3.98 | |  | 0.08 | |  |  |
|  |  |  |  |  |  |  |  |  | [0.43-35.26] | |  |  |  |  |  |  |  |  |  |  |  |  |  |  |  |  |  |  |  |  |  |  | [0.84-18.97] | |  |  |  |  |  |
|  |  |  |  |  |  |  |  |  |  |  |  |  |  |  |  |  |  |  |  |  | [0.44-35.58] | |  |  |  |  |  |  |  |  |  |  |  |  |  |  |  |  |  |

**AEs, adverse events; OR, odds ratio.**

**^a^: including nausea, vomit, diarrhea, stomatitis; ^b^: including neutropenia, febrile neutropenia, thrombocytopenia, anemia; ^c^: including neutropenia, febrile neutropenia, thrombocytopenia, anemia, nausea, vomit,**

**diarrhea, stomatitis; ^d^: reported ORs refer to *DPYD* c.1905+1G/A vs *DPYD* c.1905+1G/G carriers; ^e^: reported ORs refer to *DPYD* c.2846A/T vs *DPYD* c.2846A/A carriers; ^f^: reported ORs refer to “*DPYD* c.1905+1G/A or *DPYD* c.2846A/T” vs “*DPYD* c.1905+1G/G and *DPYD* c.2846A/A” carriers. *P* values in bold indicate statistical significance.**
