# Supplementary material for: DPYD and UGT1A1 genotyping to predict adverse events during first-line FOLFIRI or FOLFOXIRI plus bevacizumab in metastatic colorectal cancer
Source: Oncotarget. 2017 Dec 21;9(8):7859–66. doi: 10.18632/oncotarget.23559 (PMC5814264; doi:10.18632/oncotarget.23559)
Supplement: Supplementary file 3 [file oncotarget-09-7859-s003.docx]

**Supplementary Table 5. Univariate and multivariate analyses testing association hypotheses of *UGT1A1* variants with AEs.**

|  |  |  |  |  |  |  |  |  |  |  |  |  | **Univariate analyses** | | | |  |  |  |  |  |  |  |  | **Multivariate analyses^f^** | | | | |  |  |
| --- | --- | --- | --- | --- | --- | --- | --- | --- | --- | --- | --- | --- | --- | --- | --- | --- | --- | --- | --- | --- | --- | --- | --- | --- | --- | --- | --- | --- | --- | --- | --- |
|  |  |  |  |  |  |  |  |  |  |  |  |  |  |  |  |  |  |  |  |  |  |  |  |  |  |  |  |  |  |  |  |
|  | **Grade ≥3 AEs** |  |  | ***1/*1** | |  |  | ***1/*28** |  |  | ***28/*28** | |  |  | **OR^d^** |  |  | **OR^e^** |  |  |  |  |  | **OR^d^** |  |  | **OR^e^** |  |  |  |  |
|  |  |  |  | **carriers,** | |  |  | **carriers,** |  |  | **carriers,** | |  |  |  |  |  |  |  |  | ***P* value** |  |  |  |  |  |  |  |  | ***P* value** |  |
|  |  |  |  |  |  |  |  |  |  |  |  |  |  |  |  |  |  |  |  |  |  |  |  |  |  |  |  |  |  |  |  |
|  |  |  |  | **no. (%)** | |  |  | **no. (%)** |  |  | **no. (%)** | |  |  | **[95% CI]** |  |  | **[95% CI]** |  |  |  |  |  | **[95% CI]** |  |  | **[95% CI]** |  |  |  |  |
|  |  |  |  |  |  |  |  |  |  |  |  |  |  |  |  |  |  |  |  |  |  |  |  |  |  |  |  |  |  |  |  |
|  |  |  |  | **n=146** | |  |  | **n=251** |  |  | **n=39** | |  |  |  |  |  |  |  |  |  |  |  |  |  |  |  |  |  |  |  |
|  | **Nausea** | | 2 | | (1%) |  | 12 (5%) | |  | 0 | | (0%) |  | 3.02 | |  | 0.73 | |  | 0.21 | |  | 3.85 | |  | 0.68 | |  |  | 0.09 |  |
|  |  |  |  |  |  |  |  |  |  |  |  |  |  | [0.76-11.97] | |  | [0.03-16.09] | |  |  |  |  | [0.97-15.22] | |  | [0.03-13.58] | |  |  |  |  |
|  |  |  |  |  |  |  |  |  |  |  |  |  |  |  |  |  |  |  |  |  |  |  |  |  |  |  |  |  |  |  |  |
|  | **Vomit** | | 4 | | (3%) |  | 13 (5%) | |  | 1 | | (3%) |  | 1.79 | |  | 1.23 | |  | 0.56 | |  | 2.10 | |  | 1.17 | |  |  | 0.37 |  |
|  |  |  |  |  |  |  |  |  |  |  |  |  |  | [0.60-5.33] | |  | [0.18-8.26] | |  |  |  |  | [0.71-6.23] | |  | [0.18-7.56] | |  |  |  |  |
|  |  |  |  |  |  |  |  |  |  |  |  |  |  |  |  |  |  |  |  |  |  |  |  |  |  |  |  |  |  |  |  |
|  | **Diarrhea** | | 23 | | (16%) |  | 41 (16%) | |  | 1 | | (3%) |  | 1.04 | |  | 0.21 | |  | 0.17 | |  | 1.11 | |  | 0.20 | |  |  | 0.13 |  |
|  |  |  |  |  |  |  |  |  |  |  |  |  |  | [0.60-1.80] | |  | [0.04-1.13] | |  |  |  |  | [0.63-1.95] | |  | [0.04-1.09] | |  |  |  |  |
|  |  |  |  |  |  |  |  |  |  |  |  |  |  |  |  |  |  |  |  |  |  |  |  |  |  |  |  |  |  |  |  |
|  | **Stomatitis** | | 10 (7%) | | |  | 16 (6%) | |  | 4 (10%) | | |  | 0.91 | |  | 1.65 | |  | 0.58 | |  | 0.92 | |  | 1.56 | |  |  | 0.65 |  |
|  |  |  |  |  |  |  |  |  |  |  |  |  |  | [0.41-2.04] | |  | [0.51-5.34] | |  |  |  |  | [0.41-2.06] | |  | [0.48-5.09] | |  |  |  |  |
|  |  |  |  |  |  |  |  |  |  |  |  |  |  |  |  |  |  |  |  |  |  |  |  |  |  |  |  |  |  |  |  |
|  | **Neutropenia** | | 40 | | (27%) |  | 97 (39%) | |  | 23 | | (59%) |  | 1.66 | |  | 3.75 | |  | **0.001** | |  | 1.63 | |  | 4.29 | |  |  | **0.001** |  |
|  |  |  |  |  |  |  |  |  |  |  |  |  |  | [1.07-2.59] | |  | [1.80-7.80] | |  |  |  |  | [1.02-2.60] | |  | [1.97-9.32] | |  |  |  |  |
|  |  |  |  |  |  |  |  |  |  |  |  |  |  |  |  |  |  |  |  |  |  |  |  |  |  |  |  |  |  |  |  |
|  | **Febrile neutropenia** | | 10 (7%) | | |  | 19 (8%) | |  | 6 (15%) | | |  | 1.09 | |  | 2.52 | |  | 0.18 | |  | 1.08 | |  | 2.44 | |  |  | 0.20 |  |
|  |  |  |  |  |  |  |  |  |  |  |  |  |  | [0.50-2.38] | |  | [0.88-7.27] | |  |  |  |  | [0.50-2.34] | |  | [0.86-6.93] | |  |  |  |  |
|  |  |  |  |  |  |  |  |  |  |  |  |  |  |  |  |  |  |  |  |  |  |  |  |  |  |  |  |  |  |  |  |
|  | **Thrombocytopenia** | | 2 | | (1%) |  | 4 (2%) | |  | 0 | | (0%) |  | 1.05 | |  | 0.73 | |  | 0.97 | |  | 1.11 | |  | 0.74 | |  |  | 0.96 |  |
|  |  |  |  |  |  |  |  |  |  |  |  |  |  | [0.22-5.02] | |  | [0.03-16.10] | |  |  |  |  | [0.26-4.79] | |  | [0.04-12.37] | |  |  |  |  |
|  |  |  |  |  |  |  |  |  |  |  |  |  |  |  |  |  |  |  |  |  |  |  |  |  |  |  |  |  |  |  |  |
|  | **Anemia** | | 3 | | (2%) |  | 3 (1%) | |  | 0 | | (0%) |  | 0.58 | |  | 0.52 | |  | 0.75 | |  | 0.60 | |  | 0.36 | |  |  | 0.69 |  |
|  |  |  |  |  |  |  |  |  |  |  |  |  |  | [0.13-2.59] | |  | [0.03-10.62] | |  |  |  |  | [0.14-2.57] | |  | [0.02-6.77] | |  |  |  |  |
|  |  |  |  |  |  |  |  |  |  |  |  |  |  |  |  |  |  |  |  |  |  |  |  |  |  |  |  |  |  |  |  |
|  | **Overall** | | 34 | | (23%) |  | 62 (25%) | |  | 5 (13%) | | |  | 1.08 | |  | 0.52 | |  | 0.33 | |  | 1.15 | |  | 0.49 | |  |  | 0.22 |  |
|  | **gastrointestinal AEs^a^** | |  |  |  |  |  |  |  |  |  |  |  | [0.67-1.74] | |  | [0.19-1.40] | |  |  |  |  | [0.70-1.89] | |  | [0.18-1.33] | |  |  |  |  |
|  | **Overall hematological** | | 43 | | (29%) |  | 101 (40%) | |  | 23 | | (59%) |  | 1.61 | |  | 3.39 | |  | **0.003** | |  | 1.58 | |  | 3.82 | |  |  | **0.003** |  |
|  | **AEs^b^** | |  |  |  |  |  |  |  |  |  |  |  | [1.04-2.48] | |  | [1.63-7.03] | |  |  |  |  | [0.99-2.50] | |  | [1.76-8.29] | |  |  |  |  |
|  | **Overall AEs^c^** | | 62 | | (42%) |  | 136 (54%) | |  | 24 | | (62%) |  | 1.60 | |  | 2.14 | |  | **0.03** | |  | 1.64 | |  | 2.26 | |  |  | **0.03** |  |
|  |  |  |  |  |  |  |  |  |  |  |  |  |  | [1.06-2.41] | |  | [1.04-4.40] | |  |  |  |  | [1.06-2.53] | |  | [1.06-4.84] | |  |  |  |  |

**OR, odds ratio; AEs: adverse events.**

**^a^: including nausea, vomit, diarrhea, stomatitis; ^b^: including neutropenia, febrile neutropenia, thrombocytopenia, anemia; ^c^: including neutropenia, febrile neutropenia, thrombocytopenia, anemia,**

**nausea, vomit, diarrhea, stomatitis; ^d^: reported ORs refer to *UGT1A1* *1/*28 vs *UGT1A1* *1/*1 carriers; ^e^: reported ORs refer to *UGT1A1* *28/*28 vs *UGT1A1* *1/*1 carriers; ^f^: multivariate analysis adjusted for age, sex, treatment arm and ECOG PS. *P* values in bold indicate statistical significance.**
