# Supplementary material for: DPYD and UGT1A1 genotyping to predict adverse events during first-line FOLFIRI or FOLFOXIRI plus bevacizumab in metastatic colorectal cancer
Source: Oncotarget. 2017 Dec 21;9(8):7859–66. doi: 10.18632/oncotarget.23559 (PMC5814264; doi:10.18632/oncotarget.23559)
Supplement: Supplementary file 4 [file oncotarget-09-7859-s004.docx]

**Supplementary Table 6. Univariate and multivariate analyses testing association hypotheses of *DPYD* c.1905+1G>A and *DPYD* c.2846A>T and *UGT1A1* variants with AEs.**

|  |  |  |  |  |  |  | ***DPYD* c.1905+1G>A and *DPYD* c.2846A>T and *UGT1A1*^d^** | | | | | | | |  |  |  |  |  |  |
| --- | --- | --- | --- | --- | --- | --- | --- | --- | --- | --- | --- | --- | --- | --- | --- | --- | --- | --- | --- | --- |
|  |  |  |  |  |  |  | **Univariate analyses^d^** | | |  |  |  |  |  |  | **Multivariate analyses^d, e^** | | | |  |
|  | **Grade ≥3 AEs** |  |  | ***DPYD* c.1905+1G/G and** |  |  | ***DPYD* c.1905+1G/A or *DPYD*** |  |  |  |  |  |  |  |  |  |  |  |  |  |
|  |  |  |  |  |  |  | **c.2846A/T and/or** |  |  |  |  |  |  |  |  |  |  |  |  |  |
|  |  |  |  | ***DPYD* c.2846A/A and** |  |  |  |  |  | **OR** |  |  |  |  |  | **OR** |  |  |  |  |
|  |  |  |  |  |  |  |  |  |  |  |  |  |  |  |  |  |  |  |  |  |
|  |  |  |  |  |  |  | ***UGT1A1**28/*28 carriers,** |  |  |  |  |  | ***P* value** |  |  |  |  |  | ***P* value** |  |
|  |  |  |  | ***UGT1A1**1/- carriers, no. (%)** |  |  |  |  |  | **[95% CI]** |  |  |  |  |  | **[95% CI]** |  |  |  |  |
|  |  |  |  |  |  |  | **no. (%)** |  |  |  |  |  |  |  |  |  |  |  |  |  |
|  |  |  |  | **n=387** |  |  |  |  |  |  |  |  |  |  |  |  |  |  |  |  |
|  |  |  |  |  |  |  | **n=48** |  |  |  |  |  |  |  |  |  |  |  |  |  |
|  |  |  |  |  |  |  |  |  |  |  |  |  |  |  |  |  |  |  |  |  |
|  | **Nausea** | | 14 (4%) | |  | 0 (0%) | |  |  | 0.27 |  | 0.36 | |  | 0.24 | |  | 0.31 | |  |
|  |  |  |  |  |  |  |  |  |  | [0.02-4.65] |  |  |  |  | [0.02-3.75] | |  |  |  |  |
|  |  |  |  |  |  |  |  |  |  |  |  |  |  |  |  |  |  |  |  |  |
|  | **Vomit** | | 17 (4%) | |  | 1 (2%) | |  |  | 0.67 |  | 0.64 | |  | 0.63 | |  | 0.59 | |  |
|  |  |  |  |  |  |  |  |  |  | [0.12-3.70] |  |  |  |  | [0.12-3.35] | |  |  |  |  |
|  |  |  |  |  |  |  |  |  |  |  |  |  |  |  |  |  |  |  |  |  |
|  | **Diarrhea** | | 62 (16%) | |  | 3 (6%) | |  |  | 0.35 |  | 0.09 | |  | 0.39 | |  | 0.11 | |  |
|  |  |  |  |  |  |  |  |  |  | [0.11-1.16] |  |  |  |  | [0.13-1.22] | |  |  |  |  |
|  |  |  |  |  |  |  |  |  |  |  |  |  |  |  |  |  |  |  |  |  |
|  | **Stomatitis** | | 22 (6%) | |  | 8 (17%) | |  |  | 3.32 |  | **0.007** | |  | 3.48 | |  | **0.005** | |  |
|  |  |  |  |  |  |  |  |  |  | [1.39-7.94] |  |  |  |  | [1.45-8.36] | |  |  |  |  |
|  |  |  |  |  |  |  |  |  |  |  |  |  |  |  |  |  |  |  |  |  |
|  | **Neutropenia** | | 131 (34%) | |  | 29 (60%) | |  |  | 2.98 |  | **<0.001** | |  | 3.43 | |  | **<0.001** | |  |
|  |  |  |  |  |  |  |  |  |  | [1.61-5.52] |  |  |  |  | [1.79-6.60] | |  |  |  |  |
|  |  |  |  |  |  |  |  |  |  |  |  |  |  |  |  |  |  |  |  |  |
|  | **Febrile Neutropenia** | | 26 (7%) | |  | 8 (17%) | |  |  | 2.78 |  | **0.02** | |  | 2.83 | |  | **0.01** | |  |
|  |  |  |  |  |  |  |  |  |  | [1.18-6.54] |  |  |  |  | [1.23-6.53] | |  |  |  |  |
|  |  |  |  |  |  |  |  |  |  |  |  |  |  |  |  |  |  |  |  |  |
|  | **Thrombocytopenia** | | 5 (1%) | |  | 1 (2%) | |  |  | 1.63 |  | 0.66 | |  | 2.35 | |  | 0.32 | |  |
|  |  |  |  |  |  |  |  |  |  | [0.19-14.21] |  |  |  |  | [0.43-12.85] | |  |  |  |  |
|  |  |  |  |  |  |  |  |  |  |  |  |  |  |  |  |  |  |  |  |  |
|  | **Anemia** | | 6 (2%) | |  | 0 (0%) | |  |  | 0.61 |  | 0.74 | |  | 0.45 | |  | 0.58 | |  |
|  |  |  |  |  |  |  |  |  |  | [0.03-11.22] |  |  |  |  | [0.03-7.54] | |  |  |  |  |
|  |  |  |  |  |  |  |  |  |  |  |  |  |  |  |  |  |  |  |  |  |
|  | **Overall gastrointestinal** | | 91 (24%) | |  | 10 (21%) | |  |  | 0.86 |  | 0.68 | |  | 0.87 | |  | 0.71 | |  |
|  | **AEs^a^** | |  |  |  |  |  |  |  | [0.41-1.79] |  |  |  |  | [0.41-1.83] | |  |  |  |  |
|  | **Overall hematological** | | 137 (35%) | |  | 29 (60%) | |  |  | 2.79 |  | **0.001** | |  | 3.20 | |  | **<0.001** | |  |
|  | **AEs^b^** | |  |  |  |  |  |  |  | [1.51-5.15] |  |  |  |  | [1.66-6.15] | |  |  |  |  |
|  | **Overall AEs^c^** | | 190 (49%) | |  | 31 (65%) | |  |  | 1.89 |  | **0.05** | |  | 2.02 | |  | **0.04** | |  |
|  |  |  |  |  |  |  |  |  |  | [1.01-3.53] |  |  |  |  | [1.05-3.87] | |  |  |  |  |

**OR, odds ratio; AEs: adverse events. ^a^: including nausea, vomit, diarrhea, stomatitis; ^b^: including neutropenia, febrile neutropenia, thrombocytopenia, anemia; ^c^: including neutropenia, febrile neutropenia, thrombocytopenia, anemia, nausea, vomit, diarrhea, stomatitis; ^d^: reported ORs refer to *DPYD* c.1905+1G/A or *DPYD* c.2846A/T and/or *UGT1A1**28/*28 vs *DPYD* c.1905+1G/G and *DPYD* c.2846A/A and *UGT1A1**1/- carriers; ^e^: multivariate analysis adjusted for age, sex, treatment arm and ECOG PS. *P* values in bold indicate statistical significance.**
